# *Mus* (*Pyromys*) *dumbara*, a new endemic species of spiny mouse (Mammalia, Rodentia, Muridae) from Sri Lanka

**DOI:** 10.3897/zookeys.1280.163907

**Published:** 2026-05-26

**Authors:** Suyama H. Boyagoda, Madhava Meegaskumbura, Kelum Manamendra-Arachchi

**Affiliations:** 1 Department of Zoology, Faculty of Science, University of Peradeniya, Peradeniya, Sri Lanka Guangxi University Nanning China; 2 College of Forestry, Guangxi Key Lab for Forest Ecology and Conservation, Guangxi University, Nanning, China University of Peradeniya Peradeniya Sri Lanka; 3 Postgraduate Institute of Archaeology, University of Kelaniya, Colombo, Sri Lanka University of Kelaniya Colombo Sri Lanka

**Keywords:** *
Coelomys
*, Knuckles Mountain range, *Mus phillipsi*, *Mus platythrix*, *Mus saxicola*, *Mus shortridgei*

## Abstract

A new species of spiny mouse, Mus (Pyromys) dumbara**sp. nov**., is described from the Dumbara (Knuckles) Mountain Range in Sri Lanka, based on an integrated assessment of external morphology, cranial characteristics, mitochondrial and nuclear DNA sequence data. This species is assigned to the subgenus *Pyromys* on the basis of two defining cranial characteristics: the presence of a supraorbital ridge and incisive foramina that extend to the mid-length of first upper molar. *Mus dumbara***sp. nov**. is characterised by a tail distinctly longer than its combined head and body length and a moderately prominent supraorbital ridge which is clearer at the junction between parietal and frontal upon the orbit. There are several other external and cranial characteristics which can be used to distinguish *M. dumbara***sp. nov**. from each *pyromys* species. Genetic analysis further confirms the distinctiveness of *M. dumbara***sp. nov**. Mitochondrial cytochrome-*b* sequences reveal deep divergence from other Sri Lankan spiny mice (*M. mayori* and *M. fernandoni*), with uncorrected pairwise genetic distances exceeding 11.7%. This level of genetic separation, combined with its distinctive morphology and geographically restricted distribution in the Dumbara valley, provides strong evidence for its status as a new species endemic to Sri Lanka.

## Introduction

The genus *Mus* comprises 42 extant species globally, classified into four subgenera: *Mus*, *Coelomys*, *Pyromys*, and *Nannomys* (Musser and Carleton 2005; Mammal Diversity Database 2025). The subgenus *Mus* comprises 14 species distributed in Eurasia, while *Nannomys* includes 19 or more morphologically conservative species found exclusively in sub-Saharan Africa (Bryja et al. 2014; Lamb et al. 2014; Mammal Diversity Database 2025). The subgenus *Coelomys* with four species and *Pyromys* with five species are restricted to south and southeast Asia (Musser and Carleton 2005; Mammal Diversity Database 2025).

Rapid radiation is believed to characterise the genus *Mus*, complicating the resolution of phylogenetic relationships among its subgenera (Chevret et al. 2005; Steppan and Schenk 2017). Efforts to clarify the relationships among subgenera continue, but molecular studies indicate unresolved lineages due to limited sampling and likely complex evolutionary histories (Catzefus and Denys 1992; Lundrigan et al. 2002; Chevret et al. 2003, 2005; Meheretu et al. 2015; Steppan and Schenk 2017).

Sri Lanka is a small island in the Indian Ocean with a land area of ca 65,610 km^2^, recognised as a global biodiversity hotspot with the Western Ghats (Myers et al. 2000; Mittermeier et al. 2004). But, due to Sri Lanka’s unique climate and varied topography, it harbours an array of flora and fauna that are endemic to the island (Bossuyt et al. 2004; Gunawardene et al. 2007). Sri Lanka’s unique biogeography is shaped by its long-term isolation from the Indian mainland, despite periodic land-bridge connections during Pleistocene glaciations (Bossuyt et al. 2004). This isolation, coupled with the island’s varied topography and climatic heterogeneity, has resulted in high levels of endemism, particularly in its wet and montane zones (Gunawardene et al. 2007). Ecological barriers, such as the intervening dry lowlands, have limited biotic exchange with the Western Ghats, enabling Sri Lankan taxa to evolve independently over millions of years (Bossuyt et al. 2004). This biogeographic context provides a framework for understanding the evolution of species on the island, where montane ecosystems play a pivotal role in promoting allopatric speciation and ecological specialisation (Meegaskumbura et al. 2019).

In Sri Lanka, seven genera of murine rodents represented by 14 species are present (Phillips 1980; Corbet and Hill 1992; Musser and Carleton 2005), of which four belong to the genus *Mus.* Among the four species of *Mus* in Sri Lanka, two are included in the nominate subgenus, Mus (Mus) musculus Linnaeus, 1758 and M. (Mus) booduga Gray, 1837, while the other two belong to *Coelomys* and *Pyromys*: Mus (Coelomys) mayori Thomas, 1915 and Mus (Pyromys) fernandoni Phillips, 1932, both species endemic to the island (Phillips 1980; Corbet and Hill 1992; Musser and Carleton 2005). *Mus musculus* and *M. booduga* are widespread throughout the country and are among the most frequently encountered species in human habitations. *Mus mayori* (Mayor’s mouse) ranges from the forests of Sri Lanka’s central highlands to amongst the undergrowth in tall, damp forests of medium altitudes throughout much of the wet zone and lower hills (Phillips 1980). *Mus fernandoni* has a distribution ranging from the island’s south-eastern dry zone to the Uva Province and mid-elevation of the central hills (Phillips 1980). Both these species have restricted distribution due to fragmentation of their habitats (IUCN 2022).

From 2003 to 2005, a survey of small mammals was conducted in Sri Lanka with the aims of documenting the diversity of shrews and rodents on the island, investigating their phylogenetic relationships, and clarifying their taxonomy. During this survey, two specimens resembling *Mus mayori* were collected from Puwakpitiya, a valley within the Dumbara (Knuckles) Mountain Range. Detailed morphological and molecular analyses of these specimens provide the basis for the description of a new species, *Mus dumbara*, presented here.

## Materials and methods

**Figure 1.**
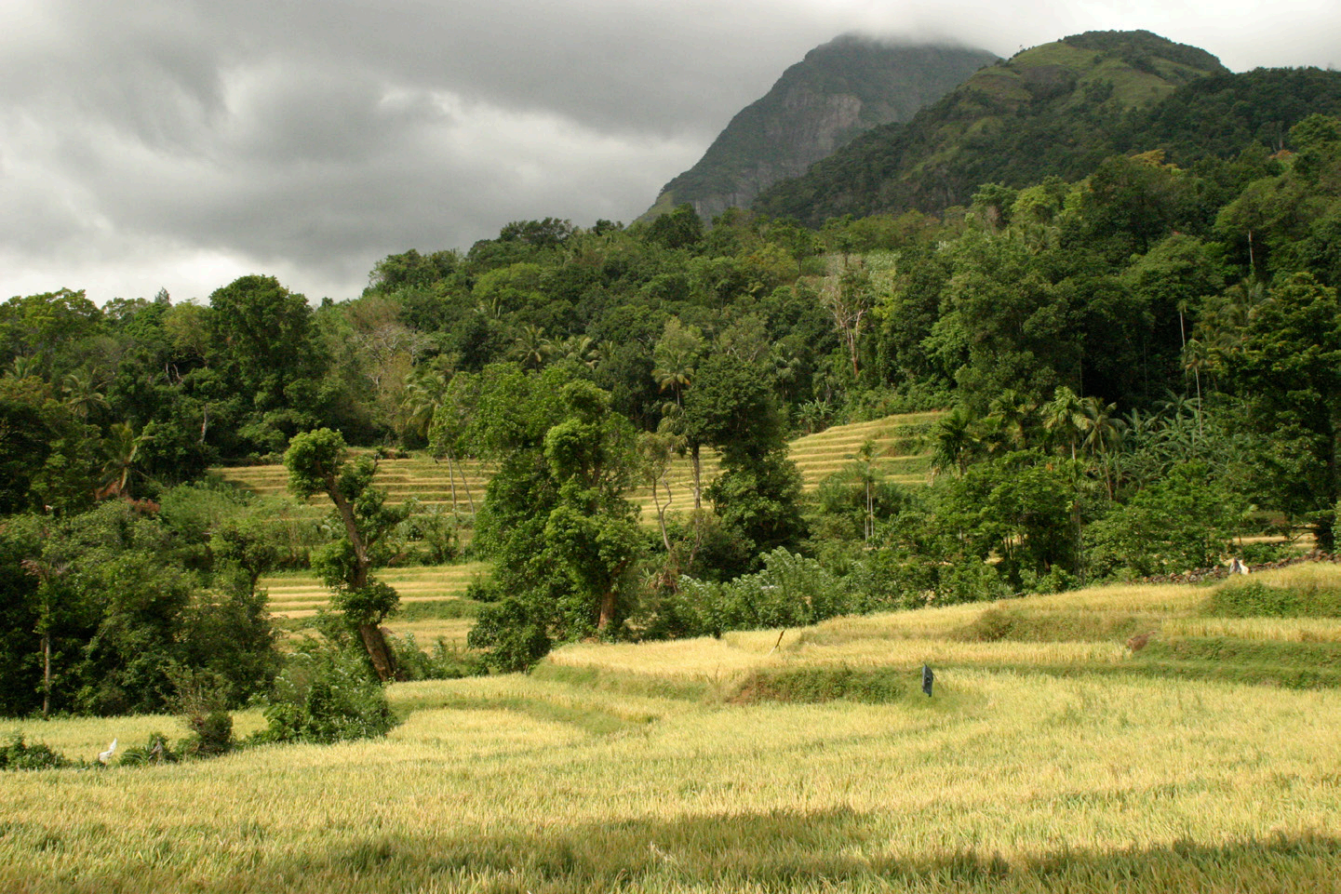
Type locality of *Mus dumbara* at Puwakpitiya, a forest edge adjacent to a paddy field.

**Figure 2.**
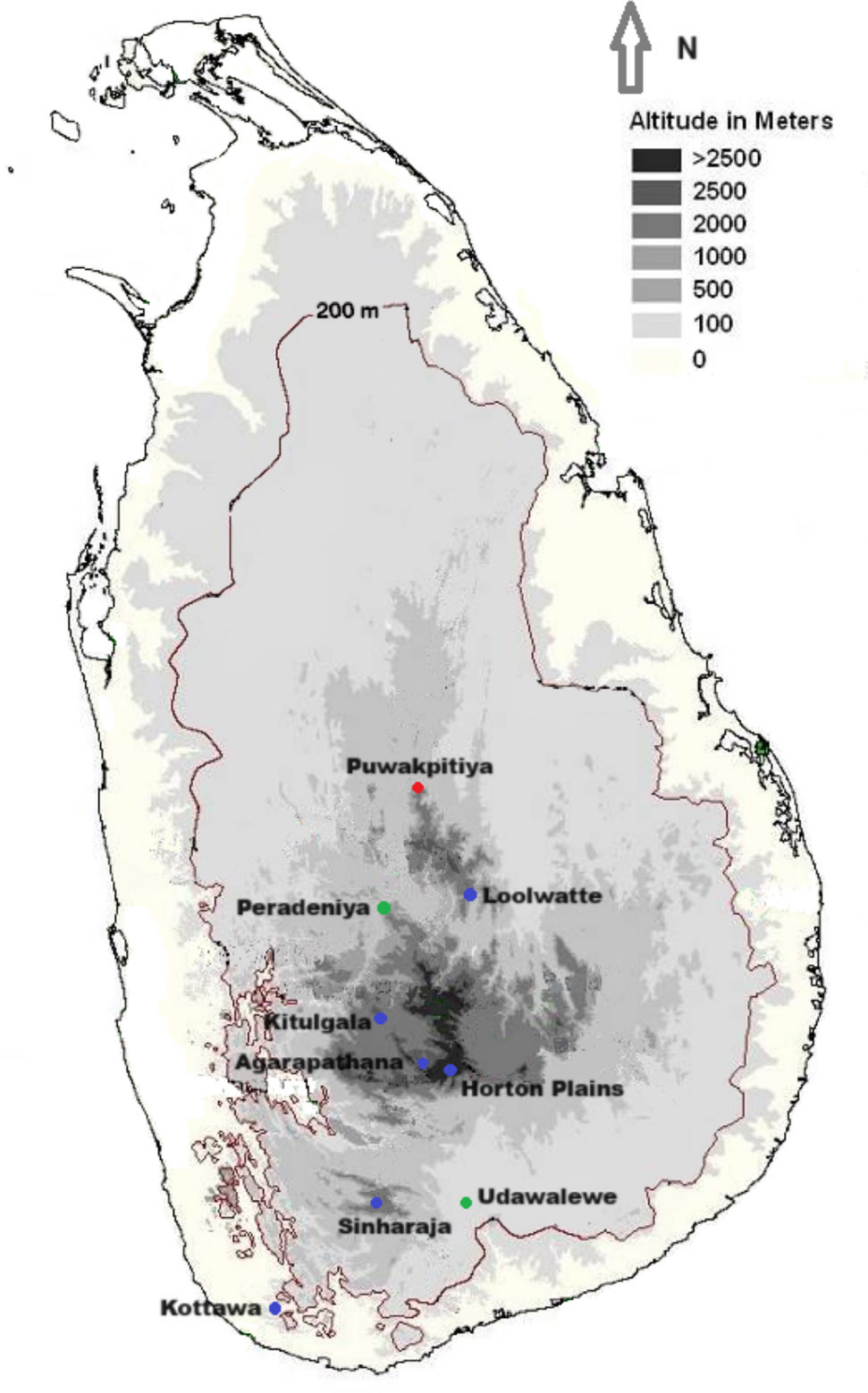
Collection sites of three spiny mice from Sri Lanka. *Mus dumbara* (red dot), *M. fernandoni* (green dots), and *M. mayori* (blue dots).

Two specimens were collected in a forest surrounding a paddy field in Puwakpitiya (07°34.73'N, 80°44.18'E, elevation 370 m; Fig. 1), a valley in Dumbara Mountain Range (Fig. 2), Matale District. Unbaited pitfall traps set between 45-cm high drift fences ~ 30–50-m long and 100 Sherman traps baited with pieces of roasted coconut were used for trapping rodents. The first specimen was trapped in a pitfall while the second was captured using a Sherman trap. Efforts to collect more specimens in 2014 and 2015 were not successful despite the investment of 1900 trap nights employing the same trapping methods. A muscle tissue sample was preserved in 90% ethanol for DNA extraction. Skulls were removed, boiled in water, cleaned with fine forceps and toothbrush, and dried. Skins were dried and preserved. Both holotype and the paratype of the new species are deposited in the National Museum of Sri Lanka (2026.01.01NH and 2026.01.02NH, respectively).

DNA was extracted from ethanol-preserved tissues using Qiagen tissue extraction kits following manufacturer’s protocols. Two mitochondrial gene fragments, cytochrome-*b* (cyt-*b*), 16S ribosomal RNA (16S) and one nuclear gene fragment, recombination activating gene 1 (Rag1) were sequenced for both individuals of the new species. DNA was amplified by PCR using 25 μl reactions containing 12.5 μl of c. 5 ng/μl template DNA, 1.25 μl of each primer (10 μM), 2.5 μl of 10 mM dNTPs, 2.5 μl of 25 mM MgCl_2_, 2.5 μl of 10× PCR buffer, and 0.625 U of Taq DNA Polymerase. PCR thermal cycle for the cyt-*b* fragment (1140 bp) was as follows: 35 cycles of denaturation at 94 °C for 30 s, annealing at 45 °C for 30 s, and extension at 72 °C for 1 min, with a final extension of 72 °C for 5 min. The same conditions were used to amplify 16S (538–544 bp), except that the annealing temperature was 48 °C. cyt-*b* and 16S were amplified using the primers MVZ 05/MVZ 14 and 16S ar/16S br, respectively. Primer sequences are as follows: MVZ 05 5’ CGA AGC TTG ATA TGA AAA ACC ATC GTTG 3’; MVZ 14 5’ GGT CTT CAT CTY HGG YTT ACA AGAC 3’; 16S ar 5’ CGC CTG TTT ATC AAA AAC AT 3’; and 16S br 5’ CCG GTC TGA ACT CAG ATC ACGT 3’. PCR thermal cycle for Rag1 (819 bp) was as follows: 35 cycles of denaturation at 95 °C for 45 s, annealing at 55 °C for 45 s, and extension at 72 °C for 1 min, with a final extension of 72 °C for 5 min. Primers used for Rag1 PCR were: AmpRAG1 F 5’ AGC TGCAGY CAR TAC CAY AAR ATG TA 3’; AmpRAG1R1 5’AAC TCA GCT GCA TTK CCA ATR TCACA 3’. Sequences were cleaned using Chromas Pro 1.7.7 (Technelysium Pty. Ltd., Tewantin, QL, Australia) and phylogenies were generated under maximum likelihood criterion using MEGA-X software (Kumar et al. 2018). Hypervariable regions in 16S gene (239–281; 354–368; 379–386 base pairs) were excluded prior to conducting phylogenetic analysis using a 483 bp sequence. We used the GTR+I+G model of sequence evolution for cyt-*b* and 16S genes, Kimura 2-parameter with gamma distribution (K2P+G) model for the Rag1 and GTR+I+G for constructing concatenated tree of all three genes, which were determined as the best nucleotide substitution models in MEGA-X. A phylogenetic analysis was also conducted excluding the third codon position to test whether saturation at the third codon position of the cyt-*b* gene obscures true evolutionary relationships. To assess the robustness of the reconstructed phylogenies, bootstrap analysis was performed on 1000 replicates, using maximum likelihood. *Tatera indica*, the Indian gerbil was used as the outgroup for the phylogenetic analysis due to suggested closest relationship of Gerbillinae to Murinae (Michaux et al. 2001; Steppan et al. 2004). Uncorrected pairwise genetic distances were generated with gamma distribution with invariant sites (G+I) using the same software. All original sequences were submitted to GenBank (Table 1). Collection site details of the Sri Lankan taxa included in the study are presented in Table 1. Collection sites of the spiny mice are shown in Fig. 2. Details of sequences extracted from the GenBank for the phylogenetic analysis are given in Suppl. material 1.

**Table 1.** Collection sites, voucher numbers of Sri Lankan specimens and GenBank accession numbers of sequences. Wildlife Heritage Trust (WHT), Sri Lanka and Peradeniya Department of Zoology (PDZ).

Species	Collection site (Longitude, Latitude, Elevation)	Voucher number	GenBank accession numbers		
			Cyt-*b*	16S rRNA	Rag1
*Mus dumbara*	Puwakpitiya (07°34.73’N, 80°44.18’E, 370 m)	2026.01.01NH	OR352820	OR359282	OR405287
		2026.01.02NH	OR352821	OR359283	OR405288
*Mus mayori*	Horton Plains (6°48.44’N, 80°48.17’E, 2150 m)	PDZ 20	OR352822	OR359284	-
		PDZ 22	OR352823	-	OR405289
		PDZ 24	OR352824	-	-
		PDZ 27	OR352825	OR359285	-
		PDZ 38	OR352826	-	-
	Knuckles Corbert gap (7°22.21’N, 80°50.28’E, 980 m)	WHT6945	-	OR359286	OR405290
		WHT6947	OR352827	OR359287	-
		WHT6951	OR352828	OR359288	-
	Agarapathana (6°52.13’N, 80°43.13’E, 1380 m)	WHT6802	KY986762	KY986840	OR405291
	Sinharaja (6°24.51’N, 80°24.56’E, 900 m)	WHT6835	KY986763	KY986839	OR405292
		WHT6863	KY986765	KY986836	OR405293
	Kitulgala (06°59.27’N, 80°24.21’E, 110 m)	WHT6864	KY986764	KY986837	-
		WHT6865	KY986766	KY986838	-
		WHT6866	KY986768	KY986842	-
		WHT6867	KY986767	KY986841	-
	Kottawa, Sabaragamuwa province (6°06.00’N, 80°18.50’E, 40 m)	WHT6883	KY986770	KY986843	OR405294
*Mus fernandoni*	Peradeniya (7°14.47’N, 80°35.54’E, 490 m)	WHT6871	OR352829	KY986844	OR405295
		WHT6876	KY986798	KY986845	-
		WHT6880	KY986799	KY986846	-
		WHT6882	KY986794	KY986847	-
	Udawalewe (06°34.98’N, 80°53.62’E, 60 m)	WHT6923	KY986795	KY986848	OR405296
		WHT6929	KY986796	KY986849	-
		WHT6930	KY986797	KY986850	-
*Mus musculus*	Puwakpitiya (07°34.73’N, 80°44.18’E, 370 m)	WHT6892	KY986772	KY986857	OR405297
*Mus booduga*	Agarapathana (6°52.13’N, 80°43.13’E, 1380 m)	WHT6873	KY697998	KY673256	OR405299
	Kitulgala (06°59.27’N, 80°24.21’E, 110 m)	WHT6858	KY986782	KY986826	OR405300
*Rattus norvegicus*	Galle (6°01.49’N, 80°12.53’E, 10 m)	PDZ44	KY697996	KY673255	MN150146
*Rattus rattus*	Udawalewe (06°34.98’N, 80°53.62’E, 60 m)	WHT6926	KY697997	KY673254	MN150147
*Bandicota indica*	Galle (6°01.49’N, 80°12.53’E, 10 m)	PDZ43	KY697990	KY673247	MN150142
*Bandicota bengalensis*	Jaffna (9°43.30’N, 80°05.05’E, 10 m)	PDZ48	KY697995	KY673253	MN160096
*Tatera indica*	Giritale (8°00.12’N, 80°55.29’E, 100 m)	WHTM05	-	OR359289	-
	Yala (6°26.06’N, 81°36.18’E, 30 m)	WHT6893	-	KY986866	MN150149

**Figure 3.**
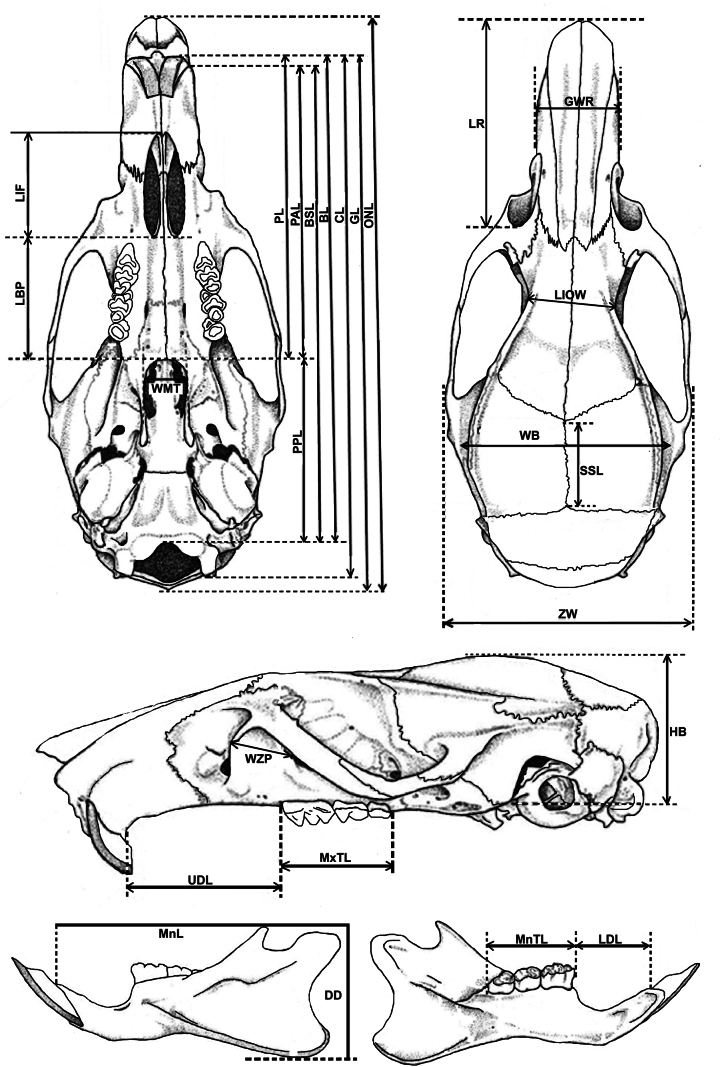
Ventral, dorsal, and lateral views of cranium, and lateral and lingual views of mandible, illustrating measurements taken in this study (figures modified from Musser et al. 2006; measurements from Buttler and Greenwood 1979; Musser et al. 2006; Meegaskumbura et al. 2007).

External and cranial measurements were taken, using Vernier callipers, to the nearest 0.1 mm. The following external measurements were taken: combined head and body length (**HBL**), head length (**HL**), tail length (**TL**), hind foot length (**HFL**), and ear height (**EH**) (Martin et al. 2011). The following cranial measurements were taken (Fig. 3): occipitonasal length (**ONL**), greatest length of skull (**GL**), condylobasal length (**CL**), basal length (**BL**), basilar length (**BSL**), post-palatal length (**PPL**), palatilar length (**PAL**), palatal length (**PL**), length of bony palate (**LBP**), length of incisive foramen (**LIF**), width of mesopterygoid fossa (**WMT**), length of rostrum (**LR**), greatest width of rostrum (**GWR**), least interorbital width (**LIOW**), width of braincase (**WB**), zygomatic width (**ZW**), sagittal suture length (**SSL**), height of braincase (**HB**), width of zygomatic plate (**WZP**), upper diastema length (**UDL**), length of maxillary tooth row (**MxTL**), depth of dentary (**DD**), mandible length (**MnL**), lower diastema length (**LDL**) and length of mandibular tooth row (**MnTL**) (Buttler and Greenwood 1979; Musser et al. 2006; Meegaskumbura et al. 2007). Skull and dental characteristics were also observed (Musser et al. 2006).

As *M. dumbara* was confirmed to belong to the subgenus *Pyromys*, it was compared with all other described *Pyromys* species from the Indian subcontinent (*M. phillipsi*, *M. platythrix*, *M. saxicola*) and Southeast Asia (*M. shortridgei*), as well as the Sri Lankan species *M. fernandoni* (Marshall 1977; Musser and Carleton 2005). In addition, it was compared with M. (Coelomys) mayori, which occurs in Sri Lanka, because its external appearance is similar to that of *M. dumbara*. Type specimens of *M. mayori* and *M. fernandoni* in the collection of the Natural History Museum, London, together with voucher specimens collected in the course of the present study were examined for comparison (Suppl. material 2).

## Results

### Phylogenetic analysis

**Figure 4.**
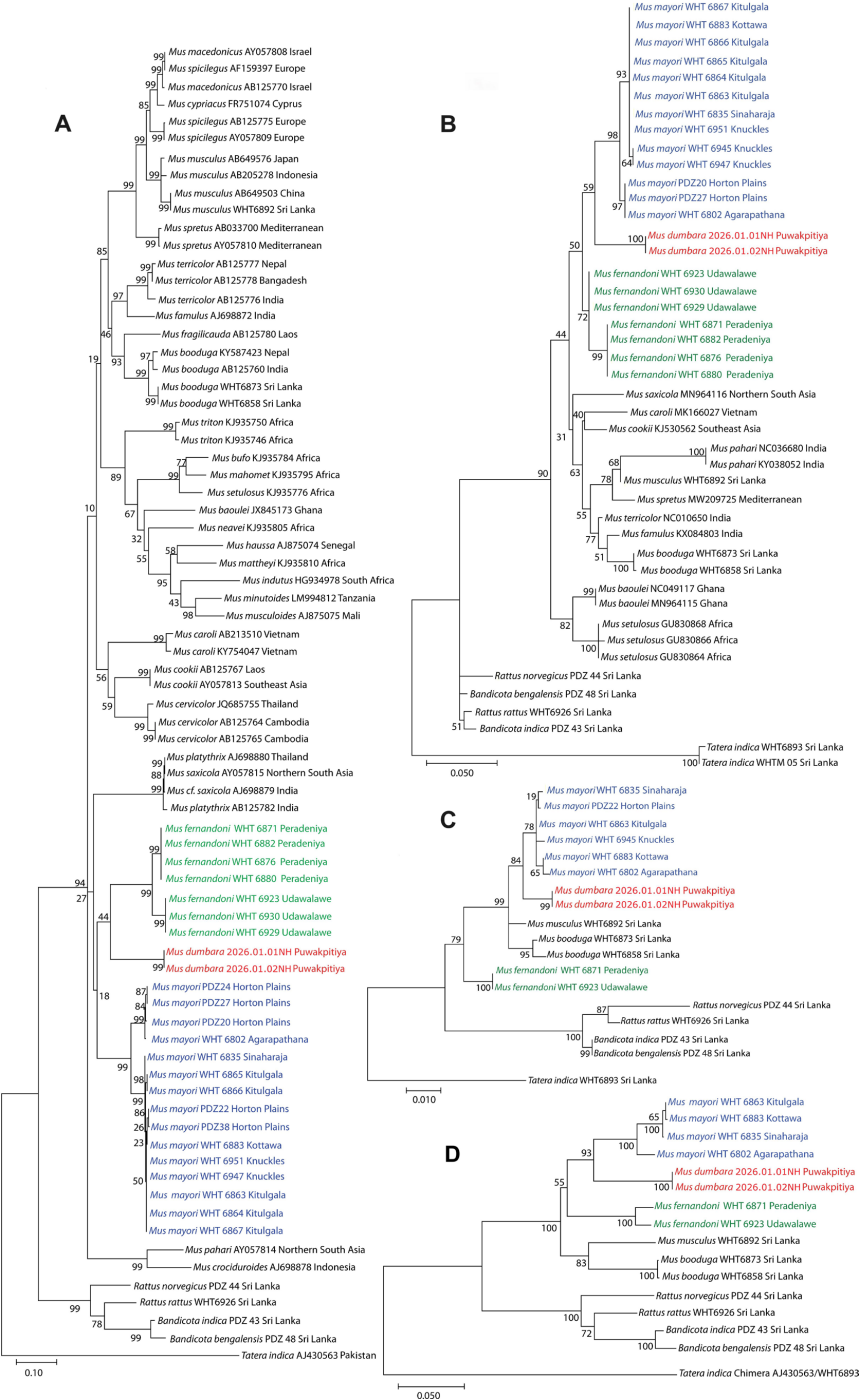
Maximum likelihood trees of (**A**) cyt-*b* gene (**B**) 16S gene (**C**) Rag1 gene (**D**) concatenated tree of all three genes with bootstrap support shown on the branches.

All species in the genus *Mus* were recovered as a monophyletic group with high bootstrap support. However, the relationships among most species that fall within the clade lacked bootstrap support (Fig. 4). *Mus dumbara* is recovered as having a weakly supported sister-species relationship to *M. fernandoni* in cyt-*b* tree and to *M. mayori* in the 16S gene tree (Fig. 4A, B, respectively). Phylogenetic analysis conducted excluding the third codon position of cyt-*b* to test the effect of saturation at third codon position resulted in a phylogeny with the same topology and similar bootstrap values as the phylogeny which resulted when all three codon positions were included (phylogeny not shown). In Rag1 tree (Fig. 4C) and concatenated tree (Fig. 4D), which included taxa only from Sri Lanka, *M. dumbara* was recovered as the sister species to *M. mayori* with 84% and 93% bootstrap support (BS), respectively. *Mus fernandoni* is placed outside the monophyletic group of *M. dumbara*, *M. mayori*, *M. musculus* and *M. booduga* having 99% BS in Rag1 gene tree. In the concatenated tree all three Sri Lankan spiny mice fell in a weakly-bootstrap supported monophyletic group (Fig. 4D). *Mus dumbara* has a percent uncorrected genetic distance of 11.78–12.54% and 12.30–12.63% for cyt-*b* gene from *M. mayori* and *M. fernandoni*, respectively (Table 2). *Mus dumbara* also shows divergence at nuclear gene level, with 1.13–1.55% and 3.11% of uncorrected genetic divergence in Rag1 gene from *M. mayori* and *M. fernandoni*, respectively. *Mus platythrix* and *M. saxicola* sequences fall within the clade of Sri Lankan spiny mice in the cyt-*b* tree but with low BS (27%). It is also noted that these two Indian species are not genetically distinct. In the 16S rRNA tree a *M. saxicola* sequence is placed outside the Sri Lankan spiny mice clade, again with weak BS.

**Table 2.** Uncorrected percent pairwise genetic distance among species in *Pyromys* and *Coelomys* subgenera.

	M. (P.) fernandoni	M. (P.) platythrix	M. (P.) saxicola	M. (C.) mayori	M. (C.) crociduroides	M. (C.) pahari
M. (P.) dumbara	12.30-12.63	14.68-15.00	14.68-14.83	11.78-12.54	15.29-15.44	15.03-15.08
M. (P.) fernandoni	-	13.42-15.26	13.42-14.65	13.07-14.47	14.39-15.26	14.47-14.91
M. (P.) platythrix	-	-	0.00-1.58	13.42-13.86	15.09-15.53	14.47-14.91
M. (P.) saxicola	-	-	-	13.42-13.86	14.91-15.09	15.26-15.44
M. (C.) mayori	-	-	-	-	14.56-14.91	14.47-15.70
M. (C.) crociduroides	-	-	-	-	-	11.58

### Taxonomic account

#### Mus dumbara sp. nov.

Taxon classification

Animalia

PhyllodocidaAphroditidae

823EDF21-81B2-5A4C-B223-2221274038B5

https://zoobank.org/F6C32390-4A1E-4792-84BF-4ECD62CAD2C4

Fig. 5A

##### Type locality.

Sri Lanka, Puwakpittiya, Matale district, 07°34.73'N, 80°44.18'E, forest edge near a paddy field, 11 March 2004.

**Figure 5.**
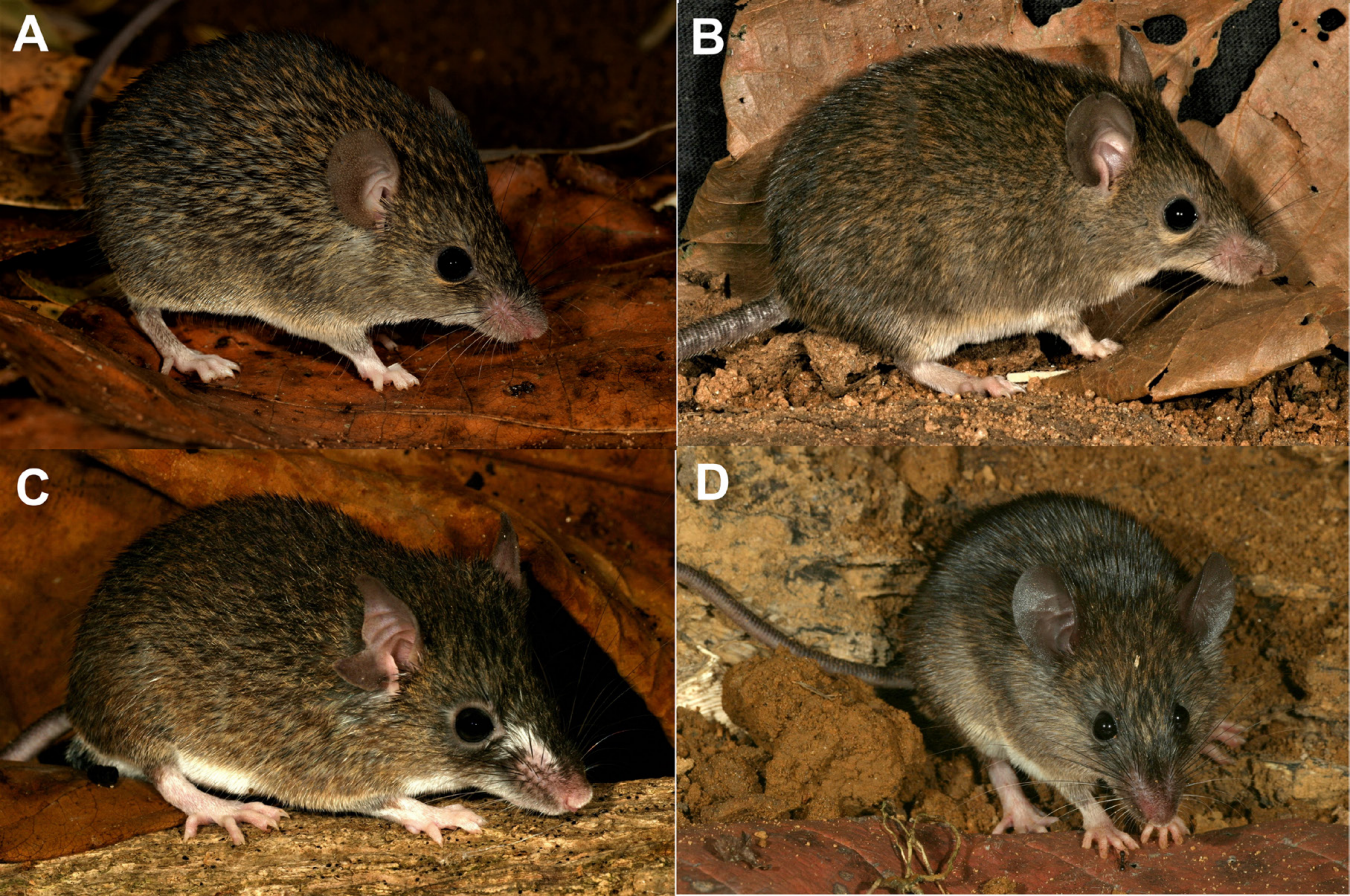
Live specimens of spiny mice from Sri Lanka showing the external similarity. **A**. The new species *Mus dumbara* (2026.01.02NH); **B**. *Mus fernandoni* (specimen not collected); **C**. *Mus mayori* (WHT 6883), a specimen with a tail having white ventral side; **D**. *Mus mayori* (WHT 6866), a specimen with a brown tail. Photographs by MM and SHB.

##### Type material.

***Holotype***. • 2026.01.01NH (female, 74.3 mm HBL), skin preserved; skull removed and preserved separately. Collected by M. M. Bahir and M. Meegaskumbura on 11 March 2004, at Puwakpittiya, Matale district (07°34.73'N, 80°44.18'E, elevation 370 m). ***Paratype***. • 2026.01.02NH (female, 77.8 mm HBL), collected by M. Meegaskumbura, M. M. Bahir and S.H. Boyagoda on 17 March 2004, other collection data same as holotype. Specimen preserved in alcohol; skull removed and preserved separately.

##### Common name.

Since the two specimens were collected from the Puwakpitiya valley in the Dumbara Mountain Range, the new species is given the common name “Dumbara valley spiny mouse” in English and “Dumbara katu heen meeya” in Sinhala.

##### Distribution.

*Mus dumbara* is currently known only from the type locality at Puwakpitiya in a valley in the north of the Dumbara Mountain Range.

##### Diagnosis.

*Mus dumbara* sp. nov. is a medium-sized species of Mus (Pyromys) characterised by a unique combination of external and cranial characters. Externally, the tail is slightly longer than the head–body length. The hind feet have pinkish-white toes, while the remainder of the dorsal surface bears a mixture of fulvous-brown, grey, and white hairs. Cranially, the species exhibits a moderately prominent supraorbital ridge, most clearly expressed at the parietal–frontal junction above the orbit. The least interorbital width is less than 4 mm. The incisive foramina extend posteriorly to the level of the mid–M1, and the difference between the lengths of the incisive foramina and the bony palate is consistently 2.2–2.7 mm. Both upper and mandibular molar cusp patterns conform to the general murine condition. On the second upper molar (M2), cusp t3 is well developed.

##### Description.

*Mus dumbara* is a medium-sized murine rodent with 74.3 and 77.8 mm HBL in the holotype and paratype, respectively, which were sexually mature females. Tail slender, its length slightly exceeding that of HBL (tail length 107.3, 104.1% of HBL) and naked. Head length 26.1, 28.0 mm. Ears prominent, with short hairs on both sides of the pinna, height 13.3, 13.5 mm. The dorsum appears speckled due to pale fulvous fine hairs and greyish brown spiny guard hairs with a broad silvery white line in the middle. The venter is white. The ventral side of tail is also white to the tip. Dorsal side of the tail is dusky. The dorsal side of fore feet and digits of hind feet are covered with white hair, giving them a pinkish white appearance. Rest of the hind feet have the same colour as the dorsum. The whiskers on the dorsal side are black with silvery white tips and the whiskers on the sides are silvery.

Skull small with an occipitonasal length of 23.4, 23.6 mm; nasals extending beyond incisors and nasal cavity, covering them in the dorsal view; rounded anterior nasal margins, narrow rostrum, premaxillaries terminating slightly anterior to the level of nasal bones at the posterior margin; moderately deep zygomatic notches; moderately prominent supraorbital ridge which is clearer at the junction between parietal and frontal upon the orbit; interparietal bone is trapezoidal forming a slight indent in the middle towards the sagittal suture; occipital is smooth and rounded; incisive foramina reaching mid first molar (M1); narrow mesopterygoid fossa of 0.7 mm, 0.7 mm; length of incisive foramina (LIF) longer than length of bony palate (LBP); difference between LIF and LBP 2.7, 2.2 mm; least interorbital width 3.5, 3.7 mm; auditory bullae prominent and globular, with short eustachian tubes; paraoccipital process small. Mandible is thin and elongated, with coronoid process that is slightly higher than the condylar process; coronoid process is small and thin; angular process is short and pointed upwards; both angular notch and sigmoid notch are shallow giving the mandible a flat broad appearance; lower diastema length slightly longer than the mandibular tooth row.

##### Comparison.

*Mus dumbara* is distinguished from *M. mayori* and *M. fernandoni*, the two Sri Lankan spiny mice, by several external and cranial characteristics. *Mus dumbara* can be differentiated from *M. mayori* by its overall smaller size (Table 3). It can be distinguished from *Mus fernandoni*, by having a tail longer than the HBL (vs the tail length shorter than the HBL in *M. fernandoni*) and pinkish white toes of hind feet with fulvous-brown, white, and grey hairs on rest of the dorsal side of the hind feet (vs pinkish white extending up to ankle on dorsal side of hind feet in *M. fernandoni*) (Fig. 5). Hind feet colour of *M. mayori* specimens vary with overall body colour. Specimens that resemble *M. fernandoni* and *M. dumbara* have pinkish-white feet, similar to *M. fernandoni* (Fig. 5C, D). In contrast, other individuals have feet that are entirely dark brown on the upper surface, or with the extreme tips of the toes appearing white.

**Table 3.** External measurements (mm) of *M. dumbara*, *M. fernandoni* and *M. mayori. M. dumbara* (*n* = 2), *M. fernandoni* (*n* = 7), *M. mayori* with long tails (*n* = 7), and *M. mayori* with short tails (*n* = 11). Average and standard deviation are given in brackets after the range of the measurements. Note: measurements of long-tailed and short-tailed *M. mayori* morphotypes are presented separately for comparison.

	*M. dumbara* (holotype, paratype)	*M. fernandoni*	*M. mayori* (long tailed)	*M. mayori* (short tailed)
Head length	26.1, 28.0	27.1-31.8 (30.4 ± 1.6)	32.2-39.4 (34.6 ± 2.7)	31.1-45.8 (34.3 ± 4.6)
Head and body length (HBL)	74.3, 77.8	83.4-90.2 (87.7 ± 2.5)	90.0-106.1 (97.1 ± 4.9)	91.7-117.5 (102.5 ± 6.9)
Tail length (TL)	79.7, 81.0	70.7-76.1 (73.7 ± 1.8)	92.0-112.1 (103.7 ± 6.9)	88.3-99.7 (93.5 ± 4.1)
(TL/HBL)×100	107.3, 104.1	81.3-87.5 (84.1 ± 2.0)	101.8-117.0 (106.9 ± 5.4)	87.1-97.6 (91.5 ± 4.8)
Ear height	13.3, 13.5	13.2-15.9 (14.7 ± 0.9)	16.0-18.1 (17.1 ± 0.9)	16.4-18.9 (17.1 ± 0.7)
Hind foot length	19.3, 18.2	18.1-19.6 (18.8 ± 0.5)	25.0-26.9 (25.8 ± 0.7)	23.9-27.5 (24.9 ± 1.0)

**Figure 6.**
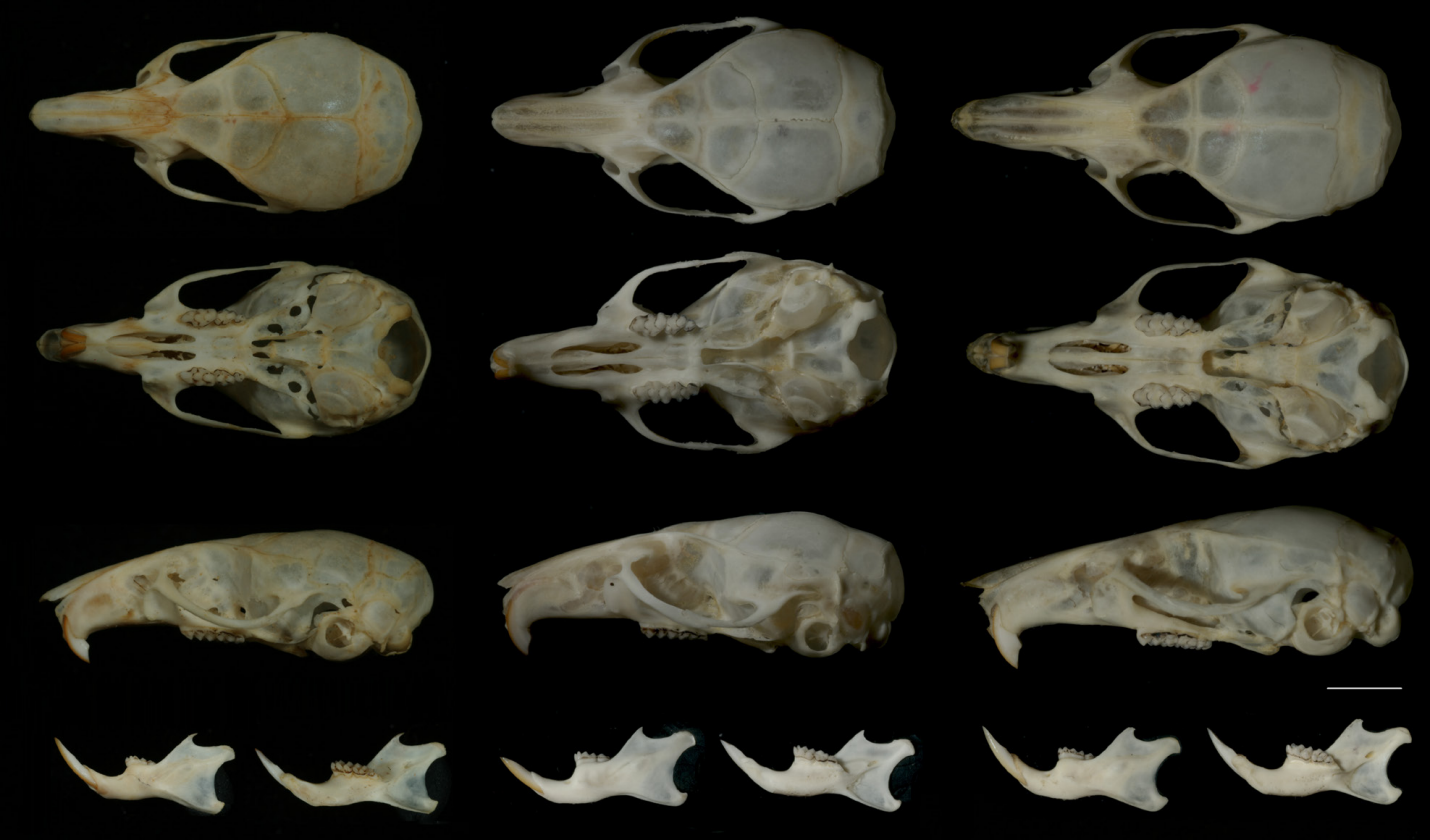
Dorsal, ventral, and lateral view of skulls and lateral and medial views of mandibles of the three species compared in the study. Left column: *Mus dumbara* (2026.01.01NH); middle column: *M. fernandoni* (WHT6923); right column: *Mus mayori* (WHT6863). Scale bar: 4 mm. Photographs by MM and SHB.

**Figure 7.**
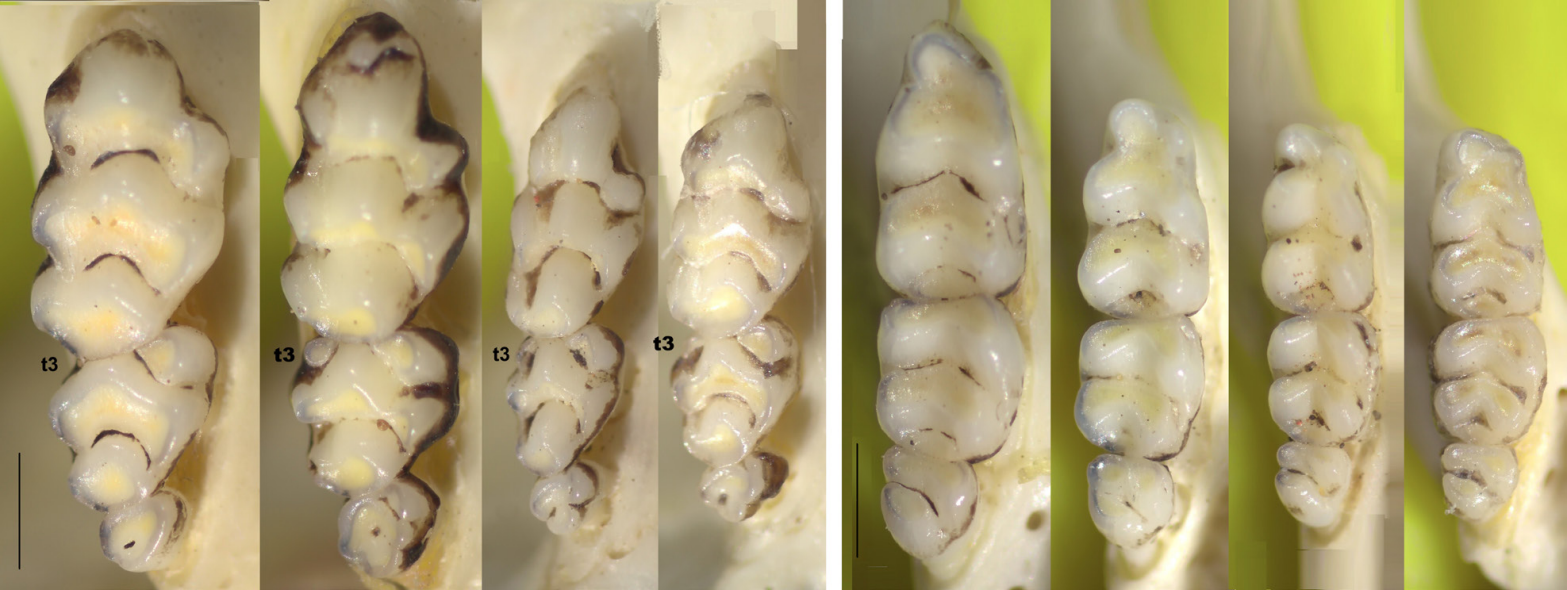
Maxillary (left) and mandibular (right) molar rows of *Mus mayori*, *M. fernandoni*, and holotype and paratype of *M. dumbara* to show the size variation and difference in t3 of M2. Voucher numbers of maxillary teeth: WHT6863, WHT6882; mandibular teeth: WHT6835, WHT6923 for *M. mayori* and *M. fernandoni* (photographs by SHB; Scale bar: 1 mm; terminology Musser et al. 2006).

Both *M. dumbara* and *M. fernandoni* belong to subgenus *Pyromys*. Hence, they share skull characteristics of *Pyromys* which can be used to distinguish them from M. (Coelomys) mayori: presence of supraorbital ridge (vs absence of supraorbital ridge in *M. mayori*); incisive foramina reaching mid M1 (vs incisive foramina terminating before anterior root of M1 in *M. mayori*) (Fig. 6). In addition to these characteristics, least interorbital width (LIOW) less than 4 mm (vs greater than 4 mm in *M. mayori*); higher difference (2.2–2.7 mm) between the length of incisive foramen (LIF) and the length of bony palate (LBP) (vs smaller difference (0.1–2.0 mm) in *M. mayori*); LIF is distinctly longer falling within the range of the LIF of *M. mayori* and as a percentage of GL, it is 23.4–25.9% (vs 19.1–22.7% in *M. mayori*); larger cusp t3 of M2 (vs very small t3 of M2 in *M. mayori*) can also be used to distinguish the two *Pyromys* species from *M. mayori* (Table 4; Fig. 7).

**Table 4.** Cranial measurements (mm) for *M. dumbara* (*n =* 2), *M. fernandoni* (*n =* 7), and *M. mayori* (*n =* 16). Mean and standard deviation are given in parentheses after the range of the measurements.

	*M. dumbara* (holotype, paratype)	*M. fernandoni*	*M. mayori*
Occipitonasal length (ONL)	23.4, 23.6	24.7-26.4 (25.8 ± 0.6)	28.4-30.8 (29.3 ± 0.7)
Greatest length of skull (GL)	22, 22.3	24.0-25.6 (24.9 ± 0.6)	27.2-29.4 (27.9 ± 0.7)
Condylobasal length (CL)	21.1, 21.5	23.3-24.9 (24.3 ± 0.5)	25.7-28.3 (26.9 ± 0.7)
Basal length (BL)	18.5, 19.0	21.1-22.4 (22.1 ± 0.6)	23.5-25.6 (24.7 ± 0.6)
Basilar length (BSL)	17.5, 17.8	20.0-21.4 (20.7 ± 0.4)	21.9-24.2 (23.0 ± 0.7)
Post-palatal length (PPL)	7.4, 8.1	8.7-9.4 (8.9 ± 0.2)	9.7-11.1 (10.4 ± 0.4)
Palatilar length (PAL)	10.1, 10.2	11.5-12.8 (12.1 ± 0.4)	12.2-13.5 (12.8 ± 0.4)
Palatal length (PL)	10.7, 10.9	12.3-13.6 (13.0 ± 0.4)	13.8-15.0 (14.4 ± 0.4)
Length of bony palate (LBP)	3.0, 3.2	3.5-3.9 (3.7 ± 0.1)	4.2-5.3 (4.7 ± 0.3)
Length of incisive foramen (LIF)	5.7, 5.4	5.9-6.5 (6.1 ± 0.2)	5.4-6.4 (6.0 ± 0.3)
LIF-LBP	2.7, 2.2	2.3-2.7 (2.4 ± 0.1)	0.1-2.0 (1.3 ± 0.5)
Width of mesopterygoid fossa (WMT)	0.7, 0.7	0.6-0.9 (0.7 ± 0.1)	1.2-1.7 (1.4 ± 0.2)
Length of rostrum (LR)	6.7, 7.2	7.7-9.0 (8.3 ± 0.5)	9.0-10.5 (9.8 ± 0.4)
LR/ONL*100	28.6, 30.5	29.7-34.1 (32.3 ± 1.5)	31.0-36.3 (33.3 ± 1.5)
Greatest width of rostrum (GWR)	2.6, 2.8	2.8-3.1 (3.0 ± 0.1)	3.1-4.2 (3.7 ± 0.3)
Least interorbital width (LIOW)	3.5, 3.7	3.7-3.9 (3.7 ± 0.1)	4.4-5.1 (4.8 ± 0.2)
Width of braincase (WB)	10.3, 10.3	10.3-10.9 (10.5 ± 0.2)	11.4-12.2 (11.8 ± 0.2)
Zygomatic width (ZW)	10.7, Broken	11.4-12.6 (12.0 ± 0.5)	12.5-13.9 (13.3 ± 0.5)
Sagittal suture length (SSL)	3.5, 3.8	3.9-4.5 (4.4 ± 0.2)	5.5-6.5 (5.9 ± 0.3)
Height of braincase (HB)	6.5, 6.9	7.0-7.7 (7.3 ± 0.3)	7.4-8.8 (8.3 ± 0.3)
Width of zygomatic plate (WZP)	1.6, 2.0	2.0-2.3 (2.1 ± 0.1)	2.3-2.8 (2.5 ± 0.2)
Upper diastema length (UDL)	5.9, 6.3	6.9-7.7 (7.3 ± 0.3)	7.4-8.8 (8.2 ± 0.4)
Length of maxillary tooth row (MxTL)	3.4, 3.4	3.8-4.3 (4.0 ± 0.2)	4.0-4.3 (4.2 ± 0.1)
Depth of dentary (DD)	5.4, 5.0	5.5-5.8 (5.8 ± 0.3)	6.0-7.2 (6.6 ± 0.4)
Mandible length (MnL)	11.9, 12.1	13.4-14.6 (14.3 ± 0.4)	15.0-16.1 (15.4 ± 0.6)
Lower diastema length (LDL)	3.5, 3.3	4.0-4.7 (4.2 ± 0.2)	3.3-4.4 (4.0 ± 0.3)
Length of mandibular tooth row (MnTL)	3.0, 3.0	3.4-3.7 (3.6 ± 0.1)	3.9-4.2 (4.1 ± 0.1)

In comparison to *M. fernandoni*, the skull of *M. dumbara* is small (Table 4). The moderately prominent supraorbital ridge which is clearer at the junction between parietal and frontal upon the orbit in *M. dumbara* is also a distinguishing feature compared to the prominent supraorbital ridge of *M. fernandoni*, along the frontal (Fig. 6). Nasals protrude further from the incisors in *M. dumbara* while they are shorter in *M. fernandoni*.

*Mus dumbara* differs from all *Pyromys* species by having a tail longer than HBL in comparison to the others having tails shorter than HBL (Bennett 1832; Wroughton 1912; Thomas 1914; Mohapatra et al. 2021). Similar to that of *M. fernandoni* all these species have prominent supraorbital ridges running along the side of parietal and frontal up to anterior margin of frontal, hence differ from supraorbital ridge of *M. dumbara*.

According to the original description, *M. shortridgei* is a relatively large mouse with a HBL of 122 mm (range 110–125); TL 101 (85–103); HFL 21.5 (20–22); EH 17.5 (17–19) (Thomas 1914). The GL of skull of type specimen is 30 mm (Thomas 1914) and Marshall (1977) reports an average skull size of 28 mm with incisive foramina long and averaging 6.7 mm. All other *Pyromys* species including *M. dumbara* are smaller than *M. shortridgei* (except some specimens of *M. platythrix*) and bears incisive foramina which are shorter than 6.5 mm.

**Figure 8.**
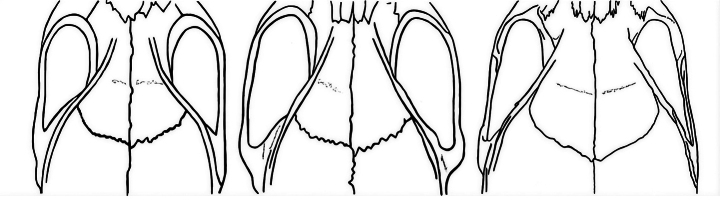
Shapes of parieto-frontal area and supraorbital ridge of *Mus phillipsi*, *M. platythrix*, and *M. dumbara*, from left to right.

*Mus phillipsi* is a small mouse, with HBL of 79.0 mm, TL of 60.0 mm, HFL of 14.0 mm, CL 21.5 mm; BSL 18 mm; WB 11 mm; UDL 6.5 mm; MxTL 3.7 mm reported for the holotype specimen (Wroughton 1912), comparable to *M. dumbara* (Tables 3, 4). It differs from *M. dumbara* by having a shorter tail in addition to its unique supraorbital ridge that diverges more abruptly when passing backwards from interorbital region that the parieto-frontal area appears ‘pyriform’ instead of evenly and gradually broadening backwards as in all other *Pyromys* species including *M. dumbara* (Fig. 8; Wroughton 1912).

**Figure 9.**
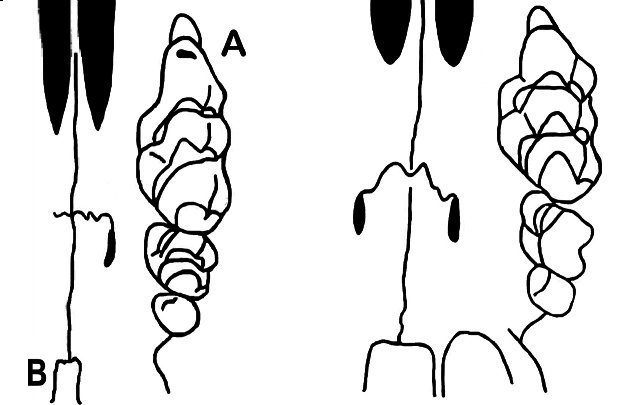
Part of the ventral view of cranium of: (on the Left) *Mus saxicola* showing the elongated anterior cusp (t2) of first upper molar (M1) with an anterior accessory cusp (**A**) and a narrow mesopterygoid fossa (**B**) in comparison to (on the Right) other *Pyromys* species with M1 lacking the elongation of anterior cusp (t2) and a broader mesopterygoid fossa (Marshall 1977).

*Mus dumbara* is distinctly different from both *M. platythrix* and *M. saxicola*. Both *M. platythrix* and *M. saxicola* are larger than *M. dumbara*. The type specimen of *M. platythrix* is medium sized with a HBL of 82.55 mm, TL of 76.2 mm, HFL of 19.05 mm and EH of 12.7 mm (Bennett 1832). However, the size range could reach as much as *M. shortridgei*, with a skull size ranging from 26–30 mm but with a shorter incisive foramina averaging 5.9 mm (Marshall 1977). *Mus saxicola* is also a medium sized rodent. Both *M. platythrix* and *M. saxicola* have elongated anterior cusp (t2) of first upper molar (M1). In addition, *M. saxicola* have an anterior accessory cusp on t2 (Fig. 9). *Mus dumbara* and other *Pyromys* species lack the elongated anterior cusp. *Mus saxicola* also has a characteristically narrow mesopterygoid fossa in comparison to broader mesopterygoid fossa in other *Pyromys* species (Fig. 9).

##### Variation.

Both specimens were of approximately the same size (HBL: 74.3, 77.8; TL: 79.7, 81.0), had the same pelage colour and texture and skull dimensions and characteristics. There is a slight difference in the extent of incisive foramina penetration between first molars in the two specimens, where 2026.01.01NH has a deeper penetration, a little beyond the first cusp of the first molar while in 2026.01.02NH, the foramina stop in the middle region of the first cusp of the first molar.

##### Etymology.

The species name dumbara is an eponym for the region it inhabits, Dumbara Mountain Range in Sri Lanka, treated here as a noun in apposition.

### Morphological key for identification of Mus (Pyromys) species

Having a small head plus body length ranging from ~50–125 mm; 2 plantar and 4 interdigital foot pads that are round, small, and smooth; first upper molar with a length exceeding half the tooth row **genus *Mus***

**Table:** 

A1	Without spiny hair	**subgenera *Mus* and *Nannomys***
A2	With spiny hair	**B**
B1	Absence of supraorbital ridge; incisive foramina terminating before anterior root of first upper molar (M1)	**subgenus *Coelomys***
B2	Presence of supraorbital ridge; incisive foramina reaching mid M1	**C**
C1	Small rodent (HBL <80 mm); tail longer than HBL; moderately prominent supraorbital ridge which is clearer at the junction between parietal and frontal upon the orbit	***Mus dumbara***
C2	Small (HBL <80 mm) to large (HBL <125 mm) rodents; tails shorter than HBL; prominent supraorbital ridges starting close behind the back of the nasals, and continue backwards across the parietals	**D**
D1	Small (HBL <80 mm) rodent; supraorbital ridge that diverges more abruptly when passing backwards from interorbital region that the parieto-frontal area appears ‘pyriform’	***Mus phillipsi***
D2	Medium to large (HBL >80–125 mm) rodents; supraorbital ridge that evenly and gradually broaden backwards	**E**
E1	Large body size (HBL 110–125 mm); incisive foramina long and averaging 6.7 mm; lack elongated anterior cusp (t2) of first upper molar (M1)	***Mus shortridgei***
E2	Medium to large (HBL >80–125 mm) rodents; incisive foramina shorter than 6.5 mm	**F**
F1	Medium to large sized (>80–125 mm) rodent; elongated t2 of M1	**G**
F2	Medium sized (>80–101 mm) rodent; lack elongated t2 of M1	***Mus fernandoni***
G1	Medium sized (>80–101 mm) rodent; Elongated t2 of M1 with an anterior accessory cusp on t2	***Mus saxicola***
G2	Medium to large (HBL >80–125 mm) rodents; Elongated t2 of M1 without anterior accessory cusp on t2	***Mus platythrix***

## Discussion

*Mus dumbara*, as described above can clearly be differentiated from currently known species in the subgenus *Pyromys* and the Sri Lankan M. (Coelomys) mayori. Phylogenetically, *M. dumbara* is placed within the monophyly of genus *Mus*, which is well established through numerous studies (Lundrigan et al. 2002; Chevret et al. 2003, 2005; Suzuki et al. 2004; Tucker et al. 2005). Due to lack of strong BS support, and incongruence among the gene trees and concatenated tree, it is not possible to make inferences regarding the evolutionary history of *M. dumbara*. Although one previous study on chromosomal phylogeny revealed a strong relationship among the four subgenera, with the first subgenus to diverge being *Coelomys*, followed by *Nannomys* at the base of a *Mus*–*Pyromys* clade (Veyrunes et al. 2006), analyses of DNA/DNA hybridization data (Catzefus and Denys 1992; Chevret et al. 2003) and phylogenetic analysis including several mitochondrial and nuclear genes (Lundrigan et al. 2002; Chevret et al. 2005; Steppan and Schenk 2017) reported unresolved relationships among the four subgenera, deduced to have resulted from rapid radiation of the genus. Incomplete taxon sampling, inconsistencies in evolution of selected DNA markers (Verneau et al. 1997, 1998), and incomplete lineage sorting are also possible reasons.

Cyt-*b*, as a highly variable protein coding gene is extensively used in phylogenetic studies and species identification. Several studies evaluating genetic divergence of cyt-*b* gene among morphologically validated rodent species of the same genus reported 2–20% or even higher pairwise uncorrected or Kimura 2-Parameter genetic divergence (Smith and Patton 1991; Johns and Avise 1998; Martin et al. 2000; Bradley and Baker 2001; Brito et al. 2022). The uncorrected pairwise genetic distance between *M. dumbara* and *M. mayori* (11.78–12.54%) and between *M. dumbara* and *M. fernandoni* (12.30–12.63%) falls well within this range.

Only a small part of the Rag1 fragment sequenced in the present study is included in Rag1 sequences used in other studies on murine rodents (Sato and Suzuki 2004; Suzuki et al. 2004). Hence, it was not possible to include Rag1 from previous studies in the analysis in order to compare genetic divergences and phylogenetic relationships among species from Sri Lanka and elsewhere. Present study revealed that *M. dumbara* has a percent uncorrected genetic distance of 1.13–1.55% and 3.11% to *M. mayori* and *M. fernandoni*, respectively. These values are comparable to pairwise Rag1 genetic distances among the other two species (*Mus booduga* and *Mus musculus*) included in the gene tree and *M. mayori* and *M. fernandoni*, which is 0.85–2.99%. Thus, morphological distinctiveness and molecular divergence provide multiple lines of evidence to support our contention that *M. dumbara* represents a distinct species of murine rodent.

*Mus platythrix* and *M. saxicola*, two of the *Pyromys* species, are also included in the cyt-*b* phylogeny, which appears genetically not distinct. Earlier studies which included these sequences reported the same (Chevret et al. 2003, 2005). The name *M. saxicola* was originally proposed explicitly as a junior synonym for *M. platythrix* by Walter Elliot in 1839, arguing that the name better expresses its habits, and as being exactly equivalent to its native name (Elliot 1839). Ellerman (1947, 1961) listed *M. saxicola* as a synonym for *M. platythrix*. However, later workers reinstated saxicola based on the species of lice found on them (Mishra et al. 1972) and distinct karyotypes (Dhanda et al. 1973). In any case, as described above *M. dumbara* is distinctly different from both species.

*Mus dumbara* is currently documented only from its type locality in Puwakpitiya, located at an elevation of 370 m within the Dumbara Mountain Range, Matale District. Both specimens were collected from a forest edge adjacent to rice paddies. The extensive small-mammal surveys conducted across Sri Lanka failed to record this species at other sites. This emphasises its potentially restricted distribution. Prior fieldwork, however, reported *M. mayori* from Gammaduwa (elevation 1067 m) in the eastern Matale Hills, west of Puwakpitiya (Phillips 1980). Furthermore, during our survey, *M. mayori* was collected from Loolwatte (elevation 980 m) within the Dumbara Mountain Range. This suggests that *M. dumbara* and *M. mayori* might occupy distinct elevationally disparate niches, with the former adapted to lower elevations and the latter more prevalent at higher altitudes.

The biogeographical patterns observed for *M. dumbara* conform to the broader ecological and evolutionary processes shaping Sri Lanka’s endemic biodiversity. As a continental island separated from the Indian mainland by the Palk Strait, Sri Lanka has experienced intermittent land-bridge connections during Pleistocene glaciations. Despite these connections, biotic interchange with southern India has been limited, likely due to ecological barriers, such as intervening dry lowlands and climatic mismatches (Bossuyt et al. 2004)​. These barriers have facilitated the independent evolutionary trajectories of montane and wet-zone species, resulting in high levels of endemism (Gunawardene et al. 2007; Meegaskumbura et al. 2019).

The Dumbara Mountain Range, a UNESCO World Heritage Site, is a distinct biogeographic unit within the Central Highlands. Spanning ca 155 km^2^, its unique topography, rising to over 1,800 m, harbours diverse ecosystems, including montane and submontane forests, grasslands, and riverine habitats (Gunatilleke et al. 2017). This topographic isolation and ecological complexity promote allopatric speciation, with species adapted to diverse ecosystems evolving independently of those in surrounding lowlands (Bossuyt et al. 2004).

While montane endemism in Sri Lanka often correlates with geographic barriers, historical climatic changes also play a critical role. The oscillations between glacial and interglacial periods influenced habitat connectivity, particularly in montane regions. During glaciations, cooler climates may have expanded montane habitats, enabling species dispersal, while interglacial warming likely re-isolated populations, reinforcing genetic and ecological divergence (Bossuyt et al. 2004; Meegaskumbura et al. 2019). Montane habitats, such as those in the Knuckles Range, act as “species pumps” and refugia, facilitating speciation while providing climatic stability during climatic oscillations. For instance, studies on shrub frogs (*Pseudophilautus*) reveal extensive diversification linked to montane isolation, ecological gradients, and allopatric divergence, with species assemblages often restricted to specific mountain ranges (Meegaskumbura et al. 2019)​. Similarly, habitat specificity and limited distribution of *M. dumbara* highlight the role of ecological isolation in promoting micro-endemism.

In the context of *M. dumbara*, the apparent absence of the species from higher altitudes or other montane ranges within Sri Lanka may be attributed to specific ecological preferences. The limited range of *M. dumbara* underscores the need for targeted studies to clarify its ecological requirements, population structure, and potential threats. As species confined to small, isolated ranges are particularly vulnerable to habitat loss and climate change and hence such studies are crucial for species conservation efforts. The biogeographic isolation of the Knuckles Mountain Range seems to have contributed to the unique evolutionary trajectory of *M. dumbara*. Future studies integrating phylogenetic, ecological, and climatic perspectives, can deepen our understanding of how montane ecosystems drive speciation and endemism of small mammals in tropical islands.

## Supplementary Material

XML Treatment for Mus dumbara

## Additional information

### Artificial Intelligence (AI) use

The authors accept full responsibility for the content of the manuscript, including the disclosure of any use of AI.

No AI tools were used in the preparation of this manuscript.

### Author ORCIDs

S. H. Boyagoda https://orcid.org/0000-0002-3555-3153

M. Meegaskumbura https://orcid.org/0000-0002-4599-6724

K. Manamendra-Arachchi https://orcid.org/0009-0009-1029-4018

### Data availability

All of the data that support the findings of this study are available in the main text or Supplementary Information.

## Supplementary materials

Supplementary material 1 GenBank accession numbers of sequences of species from other countries included in the phylogenetic study Data type docx https://binary.pensoft.net/file/1657691This dataset is made available under the Open Database License (http://opendatacommons.org/licenses/odbl/1.0/). The Open Database License (ODbL) is a license agreement intended to allow users to freely share, modify, and use this Dataset while maintaining this same freedom for others, provided that the original source and author(s) are credited. Suyama H. Boyagoda, Madhava Meegaskumbura, Kelum Manamendra-Arachchi

Supplementary material 2 Comparative material examined Data type docx https://binary.pensoft.net/file/1657692This dataset is made available under the Open Database License (http://opendatacommons.org/licenses/odbl/1.0/). The Open Database License (ODbL) is a license agreement intended to allow users to freely share, modify, and use this Dataset while maintaining this same freedom for others, provided that the original source and author(s) are credited. Suyama H. Boyagoda, Madhava Meegaskumbura, Kelum Manamendra-Arachchi
