# Supplementary material for: Mus (Pyromys) dumbara, a new endemic species of spiny mouse (Mammalia, Rodentia, Muridae) from Sri Lanka
Source: Zookeys. 2026 May 26;1280:265–85. doi: 10.3897/zookeys.1280.163907 (PMC13231179; doi:10.3897/zookeys.1280.163907)
Supplement: Supplementary material 2 — Comparative material examined [file zookeys-1280-265_article-163907__-s002.docx]

**Supplementary material 2.** Comparative material examined.

*●Coelomys mayori* [*Mus mayori mayori*], BMNH 14.12.1.7, holotype (skin and skull), Pattipola, Central Province, Ceylon [Sri Lanka], coll. Major Mayor.

*●Coelomys bicolor* [*Mus mayori pockoki*], BMNH 14.12.1.8, holotype (skin and skull), Kottowa, Sabaragamuwa Province, Ceylon [Sri Lanka], coll. Major Mayor.

*●Leggadilla fernandoni* [*Mus fernandoni*], BMNH 31.9.6.2, holotype (skin and skull), Kumbalagamuwa, Ceylon.

*●Mus mayori*, PDZ 20, PDZ 22, PDZ 23, PDZ 24, PDZ 26, PDZ 27, PDZ 29, PDZ 30, PDZ 31, PDZ 38, Horton Plains, Sri Lanka, coll. Hemamli and Boyagoda. WHT6945, WHT6947, WHT6951, Knuckles, Loolwatte, Sri Lanka, coll. Bahir and Mahamendra-Arachchi. WHT6802, Agarapathana, Sri Lanka, coll. Boyagoda, Meegaskumbura, Bahir and Nanayakkara. WHT6835, Sinharaja, Sri Lanka, coll. Meegaskumbura and Mahamendra-Arachchi. WHT6863, WHT6864, WHT6865, WHT6866, WHT6867, Kitulgala, Sri Lanka, coll. Meegaskumbura and Bahir, WHT6883, Kottawa, Sabaragamuwa province, Nanayakkara and Bahir.

*●Mus fernandoni*, WHT6871, WHT6876, WHT6880, WHT6882, Peradeniya, Sri Lanka, coll. Boyagoda, Meegaskumbura and Bahir, WHT6923, WHT6929, WHT6930, Udawalewe, Sri Lanka, coll. Bahir and Meegaskumbura.

**References**

Bryja J, Mikula O, Šumbera R, Meheretu Y, Aghová T, Lavrenchenko LA, Mazoch V, Oguge N, Mbau JS, Welegerima K, Amundala N, Colyn M, Leirs H, Verheyen E (2014) Pan-African phylogeny of *Mus* (subgenus *Nannomys*) reveals one of the most successful mammal radiations in Africa. BMC Evolutionary Biology 14(1): 1-20. https://doi.org/10.1186/s12862-014-0256-2

Cazaux B, Catalan J, Veyrunes F, Douzery EJ, Britton-Davidian J (2011) Are ribosomal DNA clusters rearrangement hotspots? A case study in the genus *Mus* (Rodentia, Muridae). BMC Evolutionary Biology 11: 1-14. <https://doi.org/10.1186/1471> -2148-11-124

Chevret P, Dobigny G (2005) Systematics and evolution of the subfamily Gerbillinae (Mammalia, Rodentia, Muridae). Molecular phylogenetics and Evolution 35(3): 674-688. <https://doi.org/10.1016/j.ympev.2005.01.001>

Chevret P, Veyrunes E, Britton-Davidian J (2005) Molecular phylogeny of the genus *Mus* (Rodentia: Murinae) based on mitochondrial and nuclear data. Biological Journal of the Linnean Society 84(3): 417-427. [https://doi.org/10.1111/j.1095- 8312.2005.00444.x](https://doi.org/10.1111/j.1095-%208312.2005.00444.x)

Chevret P, Robinson TJ, Perez J, Veyrunes F, Britton-Davidian J (2014) A Phylogeographic Survey of the Pygmy Mouse Mus minutoides in South Africa: Taxonomic and Karyotypic Inference from Cytochrome b Sequences of Museum Specimens. PLoS ONE 9(6): e98499. http://doi.org/10.1371/journal.pone.0098499

Coulibaly-N'Golo D, Allali B, Kouassi SK, Fichet-Calvet E, Becker-Ziaja B, Rieger T, Olschläger S, Dosso H, Denys C, Ter Meulen J, Akoua-Koffi C, Günther S (2011) Novel arenavirus sequences in *Hylomyscus* sp. and *Mus* (*Nannomys*) *setulosus* from Côte d'Ivoire: implications for evolution of arenaviruses in Africa. PLoS One 6(6): e20893. http://doi.org/10.1371/journal.pone.0020893

Forcina G, Camacho-Sanchez M, Tuh FY, Moreno S, Leonard JA (2021) Markers for genetic change. Heliyon 7(1): e05583. <https://doi.org/10.1016/j.heliyon.2020.e055> 83

Galan M, Pagès M, Cosson JF (2012) Next-generation sequencing for rodent barcoding: species identification from fresh, degraded and environmental samples. PLoS One 7(11): e48374. http://doi.org/10.1371/journal.pone.0048374

Kronmann KC, Nimo-Paintsil S, Guirguis F, Kronmann LC, Bonney K, Obiri-Danso K, Ampofo W, Fichet-Calvet E (2013) Two novel arenaviruses detected in pygmy mice, Ghana. Emerging Infectious Diseases, 19(11): 1832-5. <http://doi.org/10.3201> /eid1911.121491

Lee WT, Sun X, Tsai TS, Johnson JL, Gould JA, Garama DJ, Gough DJ, McKenzie M, Trounce IA, St John JC (2017) Mitochondrial DNA haplotypes induce differential patterns of DNA methylation that result in differential chromosomal gene expression patterns. Cell Death Discovery 3(1): 1-11. <http://doi.org/10.1038/cdd> iscovery.2017.62

Lundrigan BL, Jansa SA, Tucker PK (2002) Phylogenetic relationships in the genus *Mus*, based on paternally, maternally, and biparentally inherited characters. Systematic Biology 51(3): 410-431. http://doi.org/10.1080/1063515029 0069878

Martin Y, Gerlach G, Schlötterer C, Meyer A (2000) Molecular phylogeny of European muroid rodents based on complete cytochrome b sequences. Molecular phylogenetics and Evolution 16 (1): 37-47. http:/doi.org/[10.1006/mpev.1999.0760](https://doi.org/10.1006/mpev.1999.0760)

Nicolas V, Fabre PH, Bryja J, Denys C, Verheyen E, Missoup AD, Olayemi A, Katuala P, Dudu A, Colyn M, Kerbis Peterhans J, Demos T (2020) The phylogeny of the African wood mice (Muridae, Hylomyscus) based on complete mitochondrial genomes and five nuclear genes reveals their evolutionary history and undescribed diversity. Molecular Phylogenetics and Evolution 144: 106703. [https://doi.org/10. 1016/j.ympev.2019.106703](https://doi.org/10.%201016/j.ympev.2019.106703)

Pogozelski WK, Fletcher LD, Cassar CA, Dunn DA, Trounce IA, Pinkert CA (2008) The mitochondrial genome sequence of *Mus terricolor*: comparison with *Mus musculus* *domesticus* and implications for xenomitochondrial mouse modeling. Gene 418: 27-33. <https://doi.org/10.1016/j.gene.2008.04.001>

Shimada T, Aplin KP, Jogahara T, Lin LK, Herbreteau V, Gonzalez JP, Suzuki H (2007) Complex phylogeographic structuring in a continental small mammal from East Asia, the rice field mouse, *Mus caroli* (Rodentia, Muridae). Mammal Study 32(2): 49-62. <http://doi.org/10.3106/1348-6160(2007)32>

Steppan SJ, Schenk JJ (2017) Muroid rodent phylogenetics: 900-species tree reveals increasing diversification rates. PLOS One 12(8): e0183070. [https://doi.org/ 10.1371/journal. pone.0183070](https://doi.org/%2010.1371/journal.%20pone.0183070)

Suzuki H, Tsuchiya K, Takezaki N (2000) A molecular phylogenetic framework for the Ryukyu endemic rodents *Tokudaia* *osimensis* and *Diplothrix* *legata*. Molecular Phylogenetics and Evolution 5(1): 15-24. <https://doi.org/10.1006/mpev.1999.0732>

Suzuki H, Shimada T, Terashima M, Tsuchiya K, Aplin K (2004) Temporal, spatial, and ecological modes of evolution of Eurasian Mus based on mitochondrial and nuclear gene sequences. Molecular phylogenetics and evolution 33(3): 626-646. <https://doi.org/10.1016/j.ympev.2004.08.003>

Suzuki H, Nunome M, Kinoshita G, Aplin KP, Vogel P, Kryukov AP, Jin ML, Han SH, Maryanto I, Tsuchiya K, Ikeda H, Shiroishi T, Yonekawa H, Moriwaki K (2013) Evolutionary and dispersal history of Eurasian house mice Mus musculus clarified by more extensive geographic sampling of mitochondrial DNA. Heredity (Edinb). 111(5): 375-390. <https://www.nature.com/articles/hdy201360>

Terashima M, Furusawa S, Hanzawa N, Tsuchiya K, Suyanto A, Moriwaki K, Yonekawa H, Suzuki H (2006) Phylogeographic origin of Hokkaido house mice (Mus musculus) as indicated by genetic markers with maternal, paternal and biparental inheritance. Heredity (Edinb). 96 (2): 128-38. <https://www.nature.com/> articles/6800761

Tsangaras K, Wales N, Sicheritz-Pontén T, Rasmussen S, Michaux J, Ishida Y, Morand S, Kampmann ML, Gilbert MT, Greenwood AD (2014) Hybridization capture using short PCR products enriches small genomes by capturing flanking sequences (CapFlank) PLoS One 9(10): e109101. <http://doi.org/10.1371/journal.pone.0109> 101

Veyrunes F, Britton-Davidian J, Robinson TJ, Calvet E, Denys C, Chevret P (2005) Molecular phylogeny of the African pygmy mice, subgenus Nannomys (Rodentia, Murinae, Mus): implications for chromosomal evolution. Molecular Phylogenetics and Evolution 36(2): 358-69. <https://doi.org/10.1016/j.ympev.2005.02.011>

Veyrunes F, Perez J, Borremans B, Gryseels S, Richards LR, Duran A, Chevret P, Robinson TJ, Britton-Davidian J (2014) A new cytotype of the African pygmy mouse Mus minutoides in Eastern Africa. Implications for the evolution of sex-autosome translocations. Chromosome Research 22: 533-543. <https://doi.org/> 10.1007/s10577-014-9440-x

Wu HB, Liu Y (2016) The complete mitochondrial genome of the servant mouse, Mus famulus. Mitochondrial DNA Part B 1(1): 660-661. <https://doi.org/10.1080/23802> 359.2016.1219627
